# Colorimetric Sensors Based on Poly(acrylic Acid)/TiO_2_ Nanocomposite Hydrogels for Monitoring UV Radiation Exposure

**DOI:** 10.3390/gels9100797

**Published:** 2023-10-04

**Authors:** Sabina Botti, Francesca Bonfigli, Rosaria D’Amato, Jasmine Rodesi, Maria Gabriella Santonicola

**Affiliations:** 1 Fusion and Technologies for Nuclear Safety and Security Department, Photonics Micro- and Nano-Structures Laboratory, ENEA C.R. Frascati, Via E. Fermi 45, 00044 Frascati, Italy; francesca.bonfigli@enea.it (F.B.); rosaria.damato@enea.it (R.D.); 2 Department of Chemical Engineering Materials Environment, Sapienza University of Rome, Via del Castro Laurenziano 7, 00161 Rome, Italy; rodesijasmine@gmail.com

**Keywords:** colorimetric sensor, UV radiation exposure, PAA hydrogel, nanocomposite

## Abstract

In recent years, there has been an open debate on proper sun exposure to reduce the risk of developing skin cancer. The mainly encountered issue is that general guidelines for UV radiation exposure could not be effective for all skin types. The implementation of customized guidelines requires a method by which to measure the UV dose as a result of daily exposure to sunlight, ideally with an inexpensive, easy-to-read sensor. In this work, we present the characterization of nanocomposite hydrogel materials acting as colorimetric sensors upon exposure to UV light. The sensor was prepared using a poly(acrylic acid) (PAA) hydrogel matrix in which TiO_2_ nanoparticles and methylene blue (MB) were integrated. Raman mapping was used to determine the network structure of the hydrogel and its water distribution. The TiO_2_ nanoparticles dispersed in the PAA matrix maintain their photoactivity and catalyze a reaction by which methylene blue is converted into leuko-methylene. The conversion causes a discoloration effect that is visible to the naked eye and can therefore be used as an indicator of UV radiation exposure. Moreover, it was possible to tune the discoloration rate to the limit exposure of each skin type, simply by changing the ratio of titanium dioxide to dye. We obtained a response time ranging from 30 min to 1.5 h. Future work will be dedicated to the possibility of scaling up this range and to improve the sensor wearability; however, our study paves the way to the realisation of sensors suitable for public use, which could help us find a solution to the challenge of balancing sufficient UV exposure to prevent Vitamin D deficiency with excessive UV exposure that could ultimately cause skin cancer.

## 1. Introduction

Recently, there has been a debate about proper sun exposure to prevent skin cancer occurrence [1,2]. Although sunlight exposure is essential for our health as it is a key step in the production of Vitamin D, for many years, it has been well-established that excessive exposure to UV light is the primary risk factor associated with the development of skin cancer. Implementing cumulative sun exposure guidelines is a complex task since several factors must be taken into account; in fact, the intensity of UV-A rays depends on factors such as environment (e.g., presence of sand or snow, altitude), geographical position, time of day, and season, strongly affecting the cumulative time of exposure per day. In addition, there are several skin phototypes with different melanin contents that result in different sensitivities to UV radiation. The Fitzpatrick scale is an established tool that ranks skin phototypes from low to high risk of developing severe injuries after sun exposure [1]. As a consequence, sun exposure guidelines must be personalized to be truly effective, requiring measuring UV cumulative dose under daily sun exposure, using an inexpensive, easy-to-read, and affordable sensor. 

Researchers have focused on the development of devices that are compact, wearable, and capable of measuring cumulative doses. In the 1970s, Davis, Deane, and Diffey developed a polymeric sensor, based on a polysulphone film, that degraded proportionally to the accumulated dose [3]. Polysulphone would change its absorbance under UV exposure, and this variation was then measured with a spectrophotometer. This device was developed to measure the ultraviolet exposure of geriatric patients and of laboratory personnel working indoors to estimate the amount of solar radiation needed for the synthesis of vitamin D, but it was impractical for public use since it required a spectrophotometer to analyze its change in absorbance [3]. 

Over the past decade, sensors using materials that incorporate indicator systems were developed for portable use [4,5,6]. These sensors react to UV radiation by changing their color. To give a clear change in color, a UV-responsive polymer or a combination of a UV-responsive agent such as titanium dioxide (TiO_2_) and a dye were used. The tuning of the coloration/discoloration rate to match the different skin phototypes was performed just by blocking the incident light with different UV neutral density filters [4,5] or by using chemically different dyes [6]. The sensor response times span in the range of 5 min-1 h [4], 1–5 h [5], and 20–30 min [6], respectively. 

In this paper, we report a first attempt at developing a UV sensor that uses laser- synthesized TiO_2_ nanoparticles (NP) and methylene blue (MB) dye dispersed in a poly(acrylic acid) (PAA) hydrogel matrix, which is cross-linked using non-covalent bonds. Physically cross-linked hydrogels are three-dimensional networks that absorb and retain large amounts of water, from 50% to 99.5% of their own total weight [7,8]. This is possible thanks to the presence of hydrophilic groups within the network that hydrate in aqueous solution. Hydrogels are known for their biocompatibility, human-tissue-like chemical and mechanical properties, and excellent molecular permeability. They can be integrated with functional molecules and nanomaterials to achieve stimuli-responsiveness [9,10], and they are suitable for manufacturing implantable and wearable devices [11]. Raman mapping was used to determine the network structure of the PAA hydrogel and its water distribution, demonstrating that the hydrogel matrix retains its stability upon UV irradiation.

TiO_2_ is a commonly used ingredient in sunscreen formulations due to its ability to absorb UV rays, protecting not only against erythema and burns but also against the long-term effects of UV rays exposure [12,13]. During exposure to UV radiation, TiO_2_ undergoes a series of reduction–oxidation reactions that generate oxygen and hydroxyl radicals, which, in the case of MB, lead to the formation of leuko-methylene, which is white, causing a discoloration effect visible to the naked eye [14,15] that can be used as an indicator of UV radiation exposure.

We followed the discoloration process as a function of UV energy dose with both MB luminescence intensity decay and colorimetric measurements. Our results indicate that the discoloration rate can be affected by MB to TiO_2_ ratio, obtaining a sensor response time varying in the 30 min–1.5 h range.

## 2. Results and Discussion

### 2.1. Raman Confocal Spectral Imaging of PAA Network Structure

**Figure 1:**
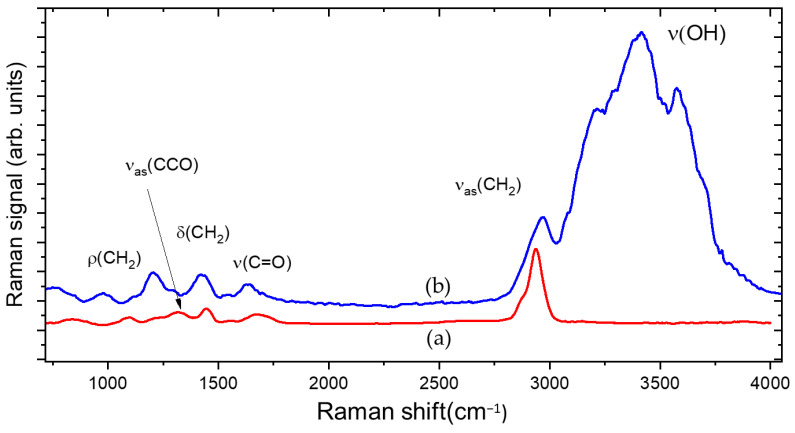
Raman spectra of PAA before hydration (a) and in gel state (b).

The PAA hydrogel was obtained by dissolving the polymer, in powder form, in distilled water. During hydration, water molecules interact with the carbonyl groups of the PAA, thus modifying the hydrogen bonds between the chains. The local dynamic of the polymer increases, and its state of aggregation changes dramatically compared to the initial state before hydration. The low consistency and the viscosity of the PAA gel were increased by neutralization with a base such as NaOH. Figure 1, curve a, shows the Raman spectrum of PAA in the dried state. Before hydration, the PAA powder has a compact structure, containing tightly coiled chains due to the hydrogen bonds between the OH group and the oxygen atom of two different carboxylic groups belonging either to two different chains or to the same chain.

Raman traces show bands associated with different possible local vibrations of the monomer, involving the carboxyl group COOH, and the groups CH_2_ and CCO. These vibrations are affected by the neighbors of the monomer. as shown in Figure 1 curve b. Every modification of the neighborhood of molecules is followed by the modification of the vibration of the molecular bonds, both after hydration and neutralization; therefore, it was possible to highlight the inclusion of water molecules in the polymeric chain [16,17]. 

In PAA, the affinity with water is determined by the presence of OH groups that behave like an electric dipole and are more susceptible to interaction with water. In the first step of hydrogel formation, the polar hydrophilic groups of the hydrogel matrix are hydrated by water, which appears in the Raman spectrum as primary bound water (3000–3200 cm^−1^ region). 

In the following step, water molecules also interact with the exposed hydrophobic groups, and these are referred to as secondary bound water (3250–3400 cm^−1^ region). Due to this interaction, the polymeric chains begin to partially uncoil and can move more freely, generating a polymeric network with large hollow spaces where additional water, absorbed during swelling, appears in the form of free water (3500–3600 cm^−1^ region) [18].

**Figure 2:**
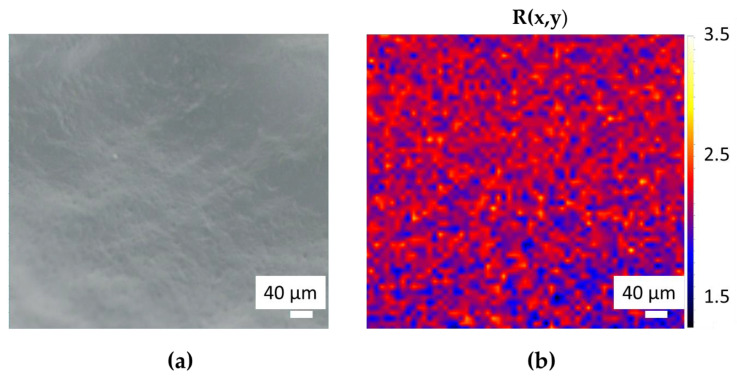
White light optical image of PAA (**a**) with the corresponding structural Raman maps (**b**).

Primary and secondary bound water together are referred to as total bound water. In the region observed with the optical microscope (Figure 2a) using white light shown in Figure 2a, we acquired a 2D Raman map composed of 3477 Raman spectra, with a step size of 10 µm. By using as a contrast parameter the ratio R between the intensity of bound and free water peaks, defined as follows,

(1) $$R=\frac{I_{bound\;water}}{I_{free\;water}},$$

we obtained the plot shown in Figure 2b. In this plot, the red color indicates the higher values of *R* corresponding to the polymeric chain, whereas the blue color indicates the areas with lower *R* values corresponding to the pores between the chain where free water is found. In this way, it was possible to highlight the structure of the PAA hydrogel, which is not visible in the white light microscope image of Figure 2a, as a network of crosslinked filaments.

From the Raman spectra, we evaluated the folding parameter R*_Folding_* of the polymer chain that is responsible for its three-dimensional structure. This parameter can be calculated as the ratio between the asymmetrical and the symmetrical portion of the CH_2_ Raman peak [19]:

(2) $$R_{Folding}=\frac{I\left(2950\;{\mathrm{cm}}^{-1}\right)}{I\left(2880\;{\mathrm{cm}}^{-1}\;\right)}.$$

For the PAA matrix, the folding parameter value is (2.5 ± 0.1). This parameter has been evaluated at each step of the hydrogel composite preparation and after UV irradiation, since variation in the folding parameter indicates changes in the hydrogel network structure that could be detrimental for its stability.

### 2.2. Inclusion of TiO_2_ Nanoparticles and MB

**Figure 3:**
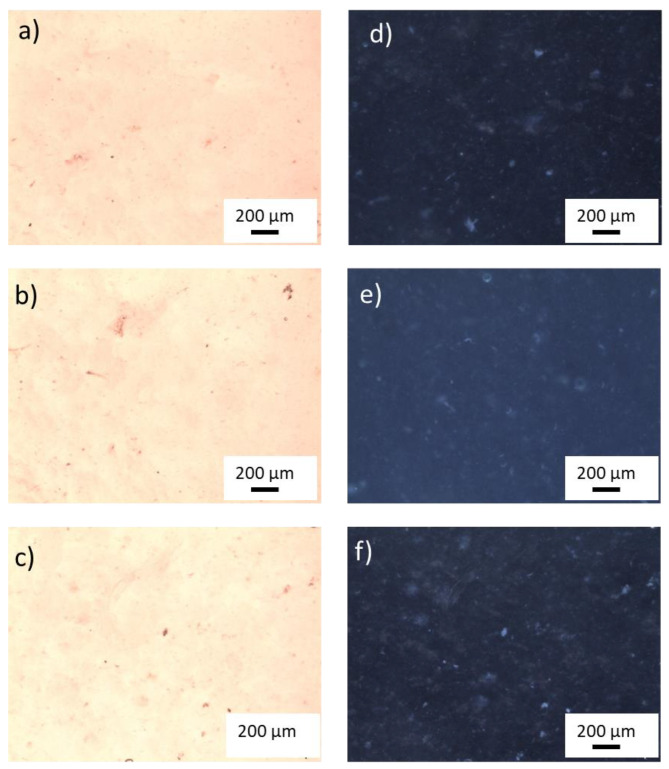
White light optical image of PAA samples with 400 ppm (**a**), 600 ppm (**b**), and 800 ppm (**c**) of TiO_2_ nanoparticles in transmission mode and the corresponding images in reflection mode (**d**–**f**). Objective 5×.

Figure 3 shows optical images, obtained using white light in transmission and reflection modes, of hydrogel samples with a content of 400, 600, 800 ppm TiO_2_ NP before the introduction of 80 ppm of MB. 

Using the software, ImageJ, a rough evaluation of TiO_2_ cluster dimensions was made. In the sample with lower titania content, the cluster dimensions are of a few tens of µm, whereas as for the optical images of the other samples, agglomerates larger than 200 µm can be observed. 

**Figure 4:**
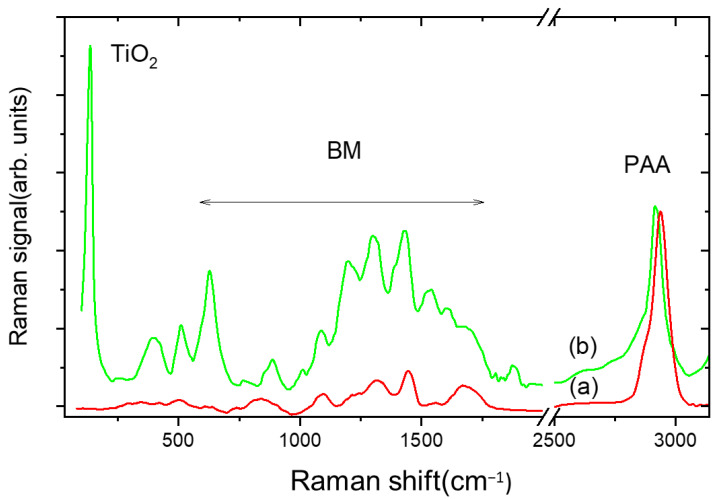
Raman spectrum of PAA hydrogel (**a**) compared with that of PAA/TiO_2_/BM composite hydrogel (**b**). TiO_2_/BM content is 400 and 80 ppm, respectively.

Figure 4 shows the comparison between the Raman trace of PAA gel (curve a) and PAA/TiO_2_/MB composite (TiO_2_ = 400 ppm, MB = 80 ppm) (curve b) in the (30–3100) cm^−1^ range. Curve b exhibits peaks with wavenumbers in the 50–300 cm^−1^ interval that are ascribed to vibrations of the laser-synthesized TiO_2_ nanoparticles in the anatase phase [20].

In the intermediate range, new intense bands appear due to the C–N–C skeletal deformation mode (517 cm^−1^), in-plane ring deformation mode of C–H (1310 cm^−1^), C–N symmetrical stretching, and C–C ring stretching (1400–1620 cm^−1^) of MB [21]. 

**Figure 5:**
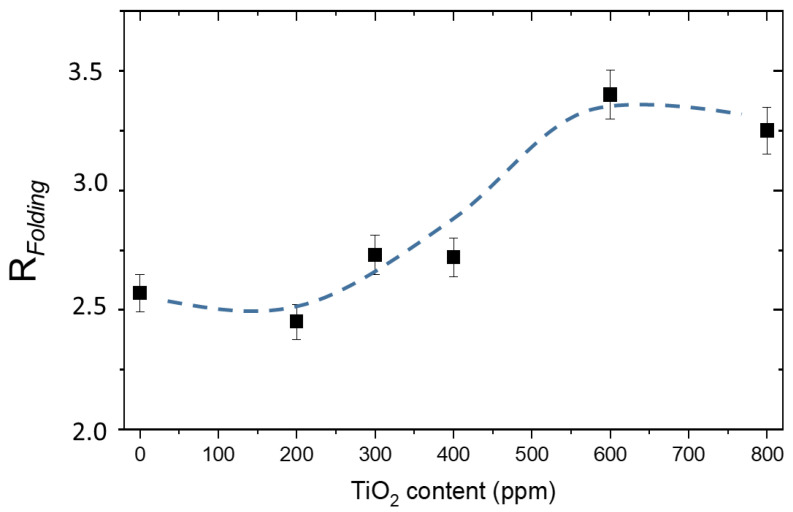
Folding parameter, R*_Folding_*, as a function of TiO_2_ content.

The folding parameter remains almost unchanged (2.6 ± 0.1) by adding 80 ppm of MB, while it increases when titania is added, as shown in Figure 5. This means that the chain becomes more compressed and folded, indicating that titania NPs are scattered in the interstices of the hydrogel lattice. 

### 2.3. Spectroscopic Study of UV Irradiation Effect on PAA/TiO_2_/MB Hydrogel

The working principle of the colorimetric sensor of this research is the UV-photocatalyzed degradation of MB. This process is carried out simultaneously by two types of chemical reactions: N- demethylation and oxidative degradation. In the demethylation process, the CH_3_ methyl groups, weak electron donors, facilitate the attack of electrophilic species such as those produced following the photoexcitation of TiO_2_ nanoparticles. N-demethylation takes place in steps, i.e., the methyl groups are removed one at a time. The color becomes less intense with each removal until it becomes white [22]. Moreover, the electrophilic species that carry out N- demethylation are responsible for reducing MB to its colorless form, leucomethylene blue. 

After 2 h of UV irradiation, the folding parameter decreases to (2.2 ± 0.1). This reduction of only 10% indicates that the hydrogel network maintains a sufficient stability upon UV exposure.

**Figure 6:**
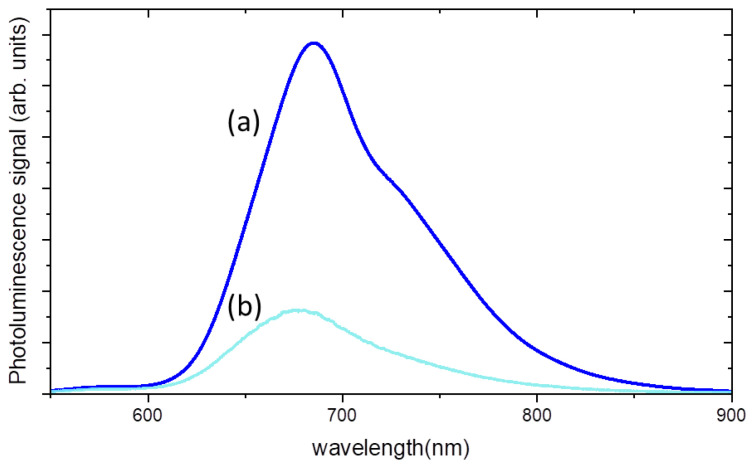
Luminescence spectra of PAA/TiO_2_/MB hydrogel composite: (**a**) before UV exposure; (**b**) after 1h of UV irradiation.

Figure 6 shows the photoluminescence spectra, excited at 532 nm, of the composite hydrogel with a titania content of 800 ppm and a MB content of 80 ppm before and after 1 h of UV irradiation, corresponding to an energy dose of 71 kJ/m^2^. A strong decrease in luminescence intensity is accompanied by a small blue shift of about 10 nm. 

These findings are a consequence of MB consumption, as it was observed that by decreasing the density of MB solutions, less re-absorption events, due to the large overlapping between absorption and emission spectra of MB, take place [23]. 

**Figure 7:**
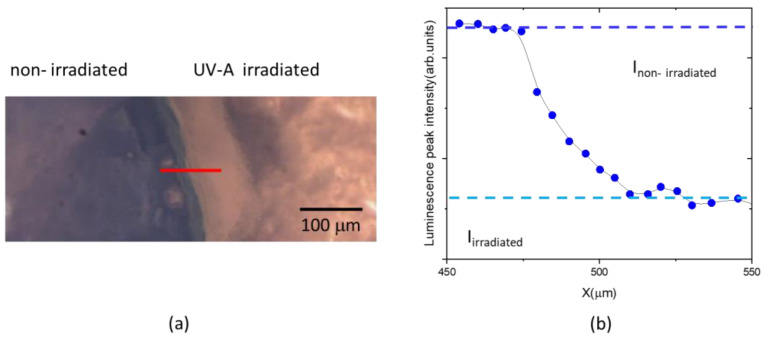
White light optical microscope image of transition region between non-irradiated and irradiated parts of PAA/TiO_2_/MB hydrogel with a TiO_2_/MB content of 800:80 ppm (**a**). Luminescence peak intensities measured along the red transition line (**b**).

The variation of luminescence peak intensity of MB is a primary index for quantifying the effect of UV-A dose on the hydrogel composite sample. To better highlight the irradiation effect, the hydrogel samples were placed under a UV-A lamp after being partially covered using aluminium foil to expose only the upper portion to UV-A. After irradiation, the aluminum foil was removed; the obtained optical image of the non-irradiated and the irradiated area of the sample is shown in Figure 7a. Luminescence spectra were acquired at each point, with a step size of 5 µm, along a line crossing both areas. The corresponding peak intensities were calculated and are shown in Figure 7b.

As expected, the luminescence peak intensity decreases along the transition line between the non-irradiated and irradiated part of sample. The initial (I_non-irradiated_) and final (I_irradiated_) values have been extrapolated (see Figure 7b).

**Figure 8:**
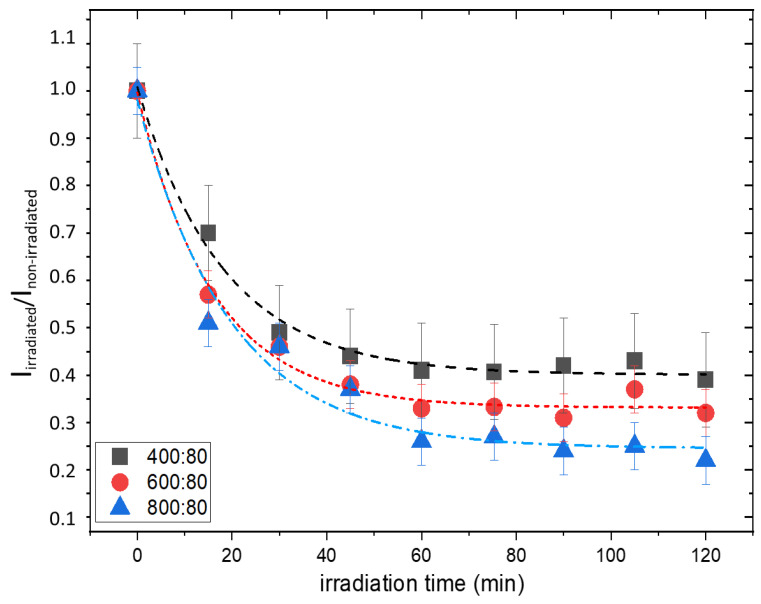
I_irradiated_/I_non- irradiated_ ratio as a function of irradiation time for the different gel formulations, with the corresponding best-fitted exponential decay curves.

To follow the kinetic of MB consumption of hydrogel composites, samples with different contents of TiO_2_ nanoparticles and the same content of MB (80 ppm) were irradiated for 2 h, with a time interval of 15 min, in which an integral dose of 18 kJ/m^2^ was released. The ratio I_irradiated_/I_non-irradiated_ was calculated at each step, and the plots, shown in Figure 8, were built. 

The curves in Figure 8 could be fitted to a first-order decay kinetic law with a rate constant which ranges in the 0.05–0.06 min^−1^ interval, corresponding to a half-time of photodegradation in the 11–14 min range. However, in the case of gel with lower titania content, an almost-steady value of the ratio I_irradiated_/I_non-irradiated_ is reached after about 45 min of irradiation, corresponding to a dose of 53 kJ/m^2^, while in the case of 800 ppm of TiO_2_, a slight decrease in intensity ratio is always observed.

### 2.4. Colorimetric Measurements

A colorimetric analysis was carried out using the “Measure” tool from the image processing software “ImageJ”. The intensities were normalized to obtain an initial null value that decreases in percentage via the following formula:

(3) $$∆\;color\;intensity\;\left(\%\right)=\left(\frac{I\left(t\right)-I_{0}}{I_{0}}\right)\times 100.$$

**Figure 9:**
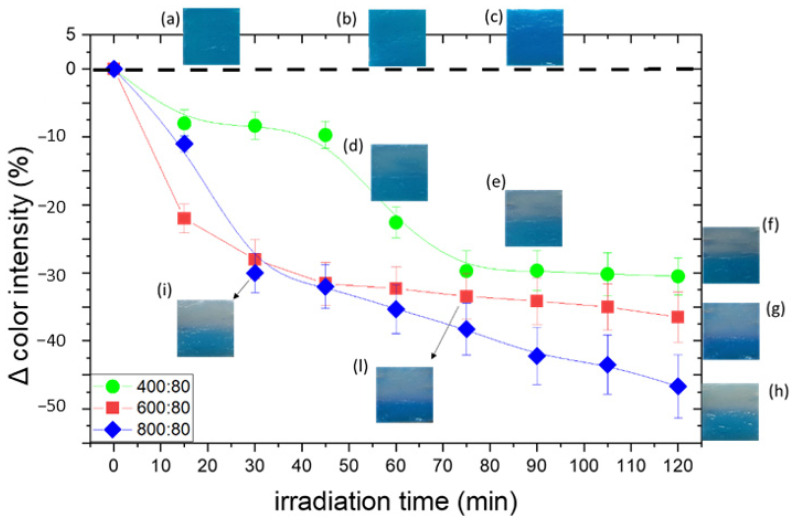
Discoloration curves of PAA/TiO_2_/MB hydrogels with different TiO_2_/MB. The insets (**a**–**c**) show the photos of non-irradiated samples; the photos in the insets (**d**–**i**,**l**) correspond to irradiated hydrogels, partially covered during irradiation.

The obtained normalized values are shown in the plots of Figure 9 as a function of irradiation time. The standard deviations are due to the positioning of the gel on a glass slide with the aid of a needleless syringe, which caused the consequently uneven surface to cast shadows that affected the measurements. In Figure 9, pictures (a, b, c) of non-irradiated PAA/TiO_2_/MB hydrogels at different TiO_2_/MB ratios are shown in the insets. The photos in the insets d-l correspond to irradiated hydrogels, partially covered during irradiation. Initial color differences are due to different concentrations of TiO_2_ that tend to bring the color towards lighter tones (Figure 9a–c). 

By changing the ratio of TiO_2_ to MB, as the content of TiO_2_ decreases, the rate of discoloration also decreases: a glaring discoloration occurs after 30, 75, and 90 min of irradiation respectively. Samples with 400 ppm TiO_2_ start fading only after a certain exposure time, exhibiting threshold behavior, i.e., the sample begins to lose color only after having absorbed a certain dose (72 kJ/m^2^, Figure 9d–f), and after 2 h of irradiation, the discoloration reaction is still not complete. This result suggests that there is a minimum threshold of TiO_2_ to dye ratio that is necessary in order to obtain the desired complete discoloration; under this threshold value, the amount of TiO_2_ is not sufficient to completely discolor the MB, most probably because the MB is only partially adsorbed onto the TiO_2_ NP surface. 

Previous studies reported that the kinetic of dye degradation in solutions depends on parameters of the solution, such as concentration of dye, pH, and so on; whereas in the solid state, these parameters do not play a significant role [5]. In our work, we observed a concentration-dependent variation in discoloration behavior because the hydrogel structure encloses in its network a certain amount of free water in which MB is diluted. To this respect, differently from other devices [4,5], this PAA/TiO_2_/MB system can be tuned to match the needs of different skin phototypes without using UV light filters.

## 3. Conclusions

In this work, a UV radiation sensor based on PAA/TiO_2_/MB composite hydrogel material has been fabricated. The intensity of the coloration assumed by the gels before and after UV-A irradiation was analyzed to demonstrate its potential utility for public use. Our study demonstrated that the titania-to-MB ratio is a key parameter that affects the discoloration rate, although it had a minor effect on the photoluminescence degradation kinetic. We tuned the sensor response time from 30 min, to give a quick alarm for sunscreen reapplication, to 1.5 h, which roughly corresponds to the maximum daily exposure time for more sensitive skin phototypes. 

Given its high water content, evaporation and shrinkage of the sensor were to be expected with light exposure. However, evaporation was not relevant when exposure times were kept in the 2-h range, meaning that the sensor is, in fact, physically stable in that time frame. 

The proposed sensor can be mounted on a bracelet, thus making it wearable, and even encapsulated in a plastic support that can retain humidity so as to further avoid shrinkage phenomena. This possibility is currently under study, but the main advantage of this sensor is that different response times can be obtained by simply changing the gel formulation. In fact, this flexibility is a crucial requisite of using UV sensors in the development and implementation of personalized UV exposure guidelines, which can help find a solution to the challenge of balancing sufficient UV exposure to prevent Vitamin D deficiency with excess UV exposure, which could lead to the development of skin cancer.

## 4. Materials and Methods

### 4.1. TiO_2_ Nanoparticles

TiO_2_ is a naturally occurring metal oxide that has the appearance of an opaque crystalline powder. TiO_2_ is a semiconductor molecule with an energy gap of 3.23 eV for anatase and 3.06 eV for rutile. When absorbing a photon of equal or greater energy than that of the band gap, TiO_2_ becomes excited, releasing electrons e- in the conduction band and leaving holes h + in the valence band.

(4) $$TiO_{2}+h\nu \to TiO_{2}\left(h_{vb}^{+},e_{cb}^{-}\right)$$

Being a semiconductor, the electron–hole pairs do not recombine immediately, and the molecule stays in the excited state for a time sufficient to allow them to interact with the chemical species adsorbed on the surface of the nanoparticles through redox reactions. The vacancies of the metal oxides have a strong oxidizing power, while the electrons in the conduction band have a strong reducing power. Free electrons interact with the surrounding oxygen and water molecules to form reactive oxygen species, including superoxide ion, hydrogen peroxide H_2_O_2_, and hydroxyl radical · OH. In turn, being unstable and reactive species, reactive oxygen species interact with neighboring matter.

(5) $$H_{2}O+h^{+}\to \cdot \;OH+H^{+}$$

(6) $$O_{2}+e^{-}\to O_{2}^{-}$$

The energy required to excite the TiO_2_ nanoparticles is quite high due to the extended band gap, and excitation is only possible by exposure to UV rays. Nanoparticles tend to agglomerate, thus decreasing the surface area and, consequently, the photocatalytic activity. In this work, TiO_2_ NP have been dispersed in aqueous solution by stirring them on a magnetic plate and a sonication. We used titania nanoparticles synthetized via CO_2_ laser-induced pyrolysis reactions in an aerosol titanium(IV) isopropoxide, following a previously published protocol [20]. In this process the reactor wall remains cold and non-reactive, assuring a high purity of TiO_2_ nano-particles. The mean powder diameter estimated by BET measurements is 20 nm. 

### 4.2. Methylene Blue

Methylene blue (MB), also known by the name of methylthioninium chloride, is an organic compound of the class of aromatic heterocyclics which originally comes as a crystalline solid that has an intense green or blue color when dissolved in aqueous solution. Once only known as a dye, today, it is widely used in histology, medicine, veterinary medicine, and chemistry. In analytical chemistry, the most common use of methylene blue is as an indicator of redox reactions; in fact, once dissolved, it is blue in an oxidizing environment and colorless in a reducing environment. A 1% solution of high purity MB (A18174, Thermo Scientific Chemicals, Waltham, MA, USA) was used in combination with the TiO_2_ NPs.

### 4.3. Preparation of Nanocomposite Gels

Carbopol^®^ Ultrez 10 (CAS n. 9003-01-4) was from Lubrizol (Wickliffe, OH, USA) and was of high purity (loss on drying ≤ 2%, heavy metals ≤ 10 ppm, residual solvents ≤ 0.45%), as reported by the manufacturer. A solution of Carbopol at 3.33 wt% in water was used for the preparation of the hydrogel matrix. Since Carbopol^®^ reaches its maximum viscosity at pH = 7, a basic solution of NaOH at 20 wt% was used as a neutralizing agent [24,25,26]. Titanium dioxide nanoparticles (d = 20 nm) produced via laser pyrolysis were used as the photoactive agent combined with methylene blue. As the first step in the development of the nanocomposite hydrogels, water solutions of titanium dioxide were prepared at 400 ppm, 600 ppm and 800 ppm. To ensure proper mixing, the solution was placed on a magnetic stirrer for 30 min, sonicated for 30 min and again stirred on a magnetic plate for 15 min. Methylene blue was added at the fixed concentration of 80 ppm and the solution placed on a magnetic stirrer for 5 min. Using a fine-mesh strainer, CBP10 was added to the solution, which was further stirred for 30 min. Finally, 260 µL NaOH were added and manually mixed to reach a neutral pH and thus the desired texture. The hydrogels were stored in polypropylene Falcon tubes, shielded from light. To graph the titration curve and, consequently, to determine the volume of NaOH needed to neutralize the polymer solutions, a Seven Compact pH meter S220 and an InLab Viscous electrode by Mettler-Toledo were used. An IKA C-MAG HS 7 digital stirring plate and an Elmasonic P sonicator (f = 37 Hz, power 100%) were used to prepare the hydrogels. All types of nanocomposite hydrogels contain a quantity of water that exceeds 95% by weight. 

### 4.4. UV-A Irradiation

We chose to irradiate the samples with UV-A light since the stratosphere filters only 5% of its total amount, and it is the UV radiation to which we are most exposed. We used a commercial UV Star lamp with a power of 36 W and an irradiance of 19.8 W/m^2^, similar to the irradiance of a sunny day in Italy in May (22 W/m^2^, 16 May 2022). For each gel formulation, samples were prepared and spread on glass slides, each of which was partially shielded from light using aluminum foil. With a maximum exposure time of 2 h, samples were retrieved every 15 min for observation. The released dose on the sample in 15 min is 18 kJ/m^2^, at the end of 2 h of irradiation, the total dose is 143 kJ/m^2^.

### 4.5. Spectroscopic Measurements

Raman and luminescence spectroscopy measurements were performed with a micro-Raman spectrometer (XploRA™ PLUS;Longjumeau, France) with 532 nm excitation wavelength and a motorized xyz stage. The Raman signal was collected in the range 50–3500 cm^−1^ with an accumulation time of 0.7 s per spectrum. Luminescence spectra were acquired for 0.1 s in the 500–900 nm range. Due to the combination with a confocal microscope, Raman/luminescence spectra can be accumulated for each point of a 1D/2D prefixed grid, through a 10× objective. All the spectra were obtained with a 10-mW laser power to avoid sample heating and damaging. The Raman/luminescence maps were obtained by plotting the intensity of the chosen peaks for each spectrum.

### 4.6. Colorimetric Measurements

The kinetics of the degradation of methylene blue color due to UV exposure was obtained by analyzing pictures with the Measure tool of the ImageJ software (version 1.53) [27]. Pictures of the samples were taken under similar lighting conditions at the following times: 0, 15, 30, 45, 60, 75, 90, 105, and 120 min. Whiteness and color intensity were measured via ImageJ, normalized, and plotted against irradiation time. The obtained values were normalized to obtain a null value before irradiation, and the color variation was expressed as a percentage.

## Data Availability

All data are available from the corresponding authors upon reasonable request.
